# Aortic root enlargement in patients undergoing mitral and aortic replacement: early outcomes in a sub-Saharan population

**DOI:** 10.3389/fcvm.2023.1239032

**Published:** 2023-10-24

**Authors:** Charles Mve Mvondo, Carole Tchokouani Djientcheu, Laurence Carole Ngo Yon, Douglas Nkomo Banga, Richard Mbele, Amos Bella Ela, Alessandro Giamberti, Alessandro Frigiola, Alain Patrick Menanga, Vincent De Paul Djientcheu, Marcelin Ngowe Ngowe

**Affiliations:** ^1^ St Elizabeth Catholic General Hospital Shisong, Cardiac Centre Shisong, Kumbo, Cameroon; ^2^ Department of Cardiothoracic and Vascular Surgery, Yaoundé General Hospital, Yaoundé, Cameroon; ^3^ Department of Surgery, Faculty of Medicine and Pharmaceutical Sciences, Douala, Cameroon; ^4^ Departement of Surgery, Faculty of Medicine and Biomedical Sciences, Yaoundé, Cameroon; ^5^ Department of Surgery, Douala General Hospital, Douala, Cameroon; ^6^ Department of Cardiac Surgery, Policlinico San Donato, Milan, Italy

**Keywords:** aortic root enlargement, valve replacement, patient–prosthesis mismatch, sub-Saharan Africa, rheumatic disease

## Abstract

**Introduction:**

Aortic root enlargement (ARE) is often required to avoid patient–prosthesis mismatch (PPM) in young patients undergoing aortic surgery, including those undergoing combined mitral and aortic valve replacement (double valve replacement, DVR). Adding ARE to DVR may increase the operative risk by extending the surgical time. Herein, we review our experience with ARE in patients who underwent DVR.

**Materials and methods:**

The medical records of 69 patients who underwent DVR at our institution between February 2008 and November 2021 were retrospectively reviewed. The patients were divided into two groups according to the ARE procedure (ARE-DVR: 25 patients; DVR: 44 patients). Descriptive and comparative analyses of demographic, clinical, and surgical data were performed.

**Results:**

Among the 69 patients who underwent DVR, 35 were women (sex ratio, 0.97). The mean age at surgery was 26.7  ±  13.9 years (range: 7–62 years). Among the 47 patients aged ≤30 years, 40.4% (19/47) were aged between 10 and 20 years, and 6.3% (3/47) were aged <10 years. Patients in the ARE-DVR group were younger (23.3 ± 12.9 years vs. 28.5 ± 14.2 years, *p *< 0.05). The New York Heart Association Class ≥III dyspnea was the most common symptom (89.9%), with no differences between the two groups. Of all the patients, 84.1% had sinus rhythm. Rheumatic disease was the most common etiology in the entire cohort (91.3%). The mean aortic annulus diameter was 20.54 mm, with smaller sizes found in the ARE-DVR group (18.00 ± 1.47 mm vs. 22.50 ± 2.35 mm, *p *< 0.05). The aortic cross-clamping duration was greater in the ARE-DVR group (177.6 ± 37.9 min vs. 148.3 ± 66.3 min, *p *= 0.047). The operative mortality rate was 5.6% for the entire cohort (ARE-DVR: 8% vs. DVR: 4.5%, *p *= 0.46). Among the patients who underwent echocardiographic control at follow-up, the mean aortic gradient was 19.6 ± 7.2 mmHg (range: 6.14–33 mmHg), with no differences among the groups.

**Conclusion:**

The association between ARE and DVR did not significantly affect operative mortality. ARE can be safely used whenever indications arise to reduce the occurrence of PPM, especially in young patients with growth potential.

## 1. Introduction

Aortic root enlargement (ARE) is a surgical technique that allows the implantation of a larger prosthesis during aortic valve replacement (AVR). Posterior ARE includes a patch enlargement of the aortic annulus through an incision extended to the anterior mitral leaflet, as first reported by Nicks et al. (1) and Manoughian and Seybold-Epting (2) more than four decades ago. These techniques are mainly recommended for children and young adults with small aortic annuli to limit the risk of patient–prosthesis mismatch (PPM), which has been associated with poor ventricular mass regression, increased risk of heart failure, and mortality (3–5). Although ARE has been shown to be a safe technique (6, 7), its association with concomitant procedures such as double valve replacement (DVR) might result in increased operative length and perioperative morbidity (8). This is particularly challenging in children and young adults with rheumatic valvular disease living in developing countries, where countless people present with multivalvular lesions (9).

This study reports our experiences with ARE in a sub-Saharan population that underwent DVR by reviewing early surgical outcomes.

## 2. Materials and methods

The clinical records of 69 patients who underwent DVR at our institution between February 2008 and November 2021 were retrospectively reviewed. A total of 44 patients (*n* = 44) underwent combined DVR and ARE, whereas 25 (*n* = 25) underwent DVR alone. Among the patients with ARE, Nick's procedure was the most common. Rheumatic heart disease was the predominant etiology in both groups. Table 1 presents the patient demographic characteristics.

**Table 1:** Patients’ demographics and preoperative characteristics.

Variables	ARE-DVR (*n* = 25)	DVR (*n* = 44)	Total	*p*-value
Age (years), mean ± SD	23.3 ± 12.9	28.5 ± 14.2	26.7 ± 13.9	<0.05
Female sex, *n* (%)	14 (56%)	21 (47.7)	35 (50.7)	<0.27
BSA (kg/m^2^), mean ± SD	1.48 ± 0.39	1.59 ± 0.28	1.55 ± 0.32	0.187
NYHA ≥III, *n* (%)	23 (92%)	39 (88.6%)	62 (89.8%)	0.68
LVEF ≤50%, *n* (%)	19 (76%)	36 (82%)	55 (79.7%)	0.28
PAPs >35 mmHg, *n* (%)	71.4%	86.1%		0.15
Aortic annulus (mm), mean ± SD	18.00 ± 1.47	22.50 ± 2.35	20.54	0.05
Aortic dysfunction, *n* (%)				
* *Pure regurgitation	21 (84%)	38 (86.3%)	59 (85.5%)	
* *Pure stenosis	1 (4%)	1 (2.2%)	2 (2.8%)	
* *Mixed	3 (12%)	5 (11.3%)	8 (11.5%)	
Mitral dysfunction, *n* (%)				
Pure regurgitation	16 (64%)	28 (63.6%)	44 (63.7%)	
Pure stenosis	4 (16%)	8 (18.1%)	12 (17.3%)	
Mixed	5 (2%)	8 (18.1%)	13 (18.8%)	
Associated TV lesions, *n* (%)	16 (64%)	23 (52.2%)	39 (56.5%)	
Atrial fibrillation, *n* (%)	2 (8%)	9 (20.4%)	11 (15.9%)	
Rheumatic etiology, *n* (%)	24 (96%)	39 (88.6%)	63 (91.3%)	

NYHA, New York Heart Association; BSA, body surface area; LVEF, left ventricle ejection fraction; PAPs, systolic pulmonary artery pressure; SD, standard deviation.

### 2.1. Preoperative evaluation and indication of ARE

The expected minimal effective orifice area (eEOA) required to avoid PPM was calculated preoperatively for all patients [eEOA = body surface area (BSA) × 0.85]. Following the analysis of the hemodynamic profiles provided by the manufacturers, prosthetic valves that provided similar or greater eEOA values were selected. ARE was performed in cases where the native aortic annulus diameter was smaller than that of the selected prosthetic valve.

### 2.2. Operative technique

**Figure 1:**
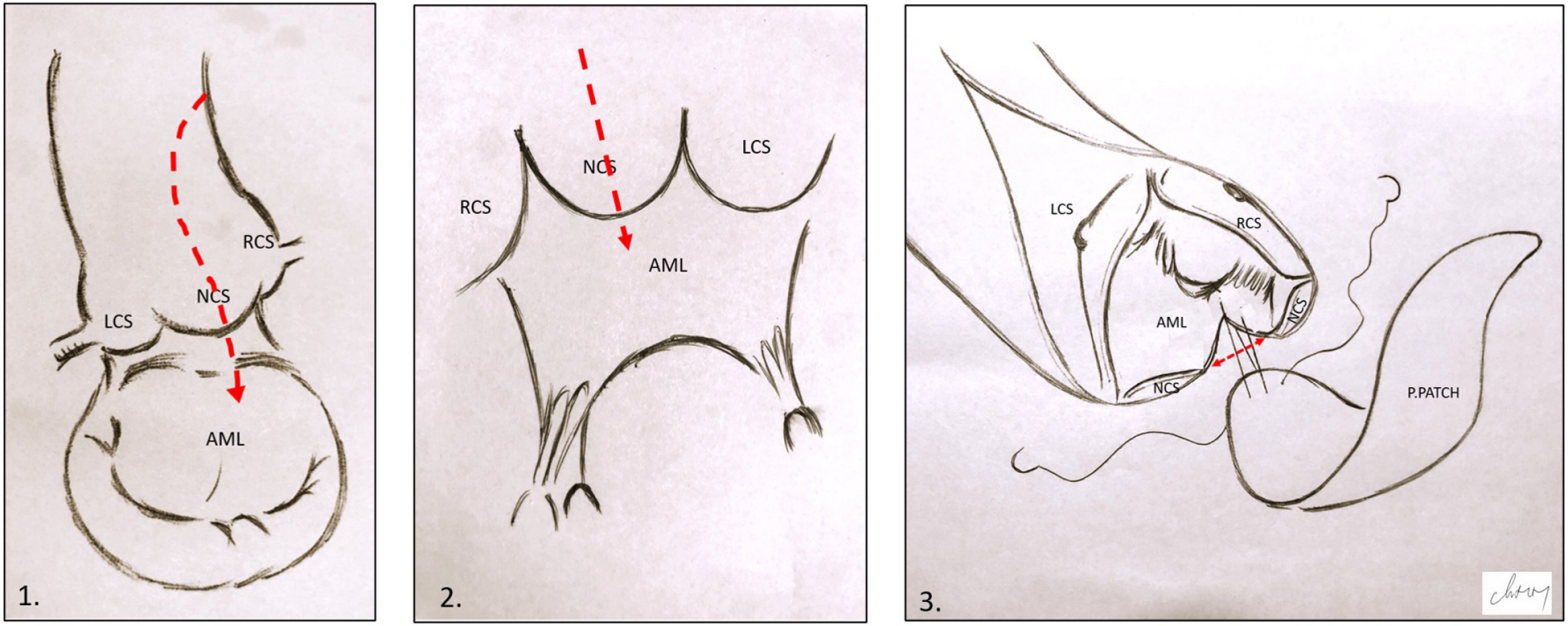
Illustration of Nick's technique. (1) and (2) Hockey stick aortotomy extended in the middle of the NCS and the anterior mitral leaflet. (3) Pericardial patch suturing; the red arrow shows the opening in the aortic annulus and AML. LCS, left coronary sinus; RCS, right coronary sinus; NCS, non-coronary sinus; AML, anterior mitral leaflet; P.PATCH, pericardial patch*.*

Following aortic cross-clamping, a cardioplegic solution was administered to the aortic root or selectively to the coronary ostia. A “hockey stick” aortotomy and complete resection of the aortic leaflets were performed, followed by annular sizing, to assess the adequacy of the selected prosthetic valve. When indicated, ARE was performed first. Our preferred technique was Nick's technique (NT) (10), using a heterologous pericardial patch through an incision in the non-coronary cusp, extending to approximately 1.5 cm into the anterior mitral leaflet (Figure 1). The annular diameter was measured after ARE to ensure proper enlargement prior to prosthetic replacement. The mitral valve was then accessed through a standard left atriotomy and replaced using single interrupted sutures reinforced with pledgets. Particular attention was paid to maintaining the annulo-ventricular continuity by preserving the posterior leaflet or implanting artificial polytetrafluoroethylene (PTFE) chords. The aortic valve was successively implanted in a supra-annular position using single interrupted sutures reinforced with pledgets.

### 2.3. Statistical analysis

Statistical analysis was conducted using SPSS version 26.0 and Microsoft Excel 2016. The variables for descriptive analysis were expressed as proportions and mean ± standard deviation, while comparative analyses were performed using the chi-squared test. The correlation between variables was established by determining the *p*-value, which was considered statistically significant at *p *< 0.05.

## 3. Results

Among the 69 patients who underwent DVR, 35 were women (sex ratio, 0.97). The mean age at surgery was 26.7 ± 13.9 years (range: 7–62 years). Among the 47 patients aged ≤30 years, 40.4% (19/47) were aged between 10 and 20 years, and 6.3% (3/47) were aged <10 years. Patients in the ARE-DVR group were younger (23.3 ± 12.9 years vs. 28.5 ± 14.2 years, *p *< 0.05). The most common symptom was New York Heart Association Class ≥III dyspnea (89.9%), with no differences between the two groups. Sinus rhythm was present in 84.1% of the patients. A left ventricular ejection fraction ≥50% was found in 77.8% of the cases. Rheumatic disease was the most common etiology in the entire cohort (91.3%). The mean aortic annulus diameter was 20.54 mm, with smaller sizes found in the ARE-DVR group (18.00 ± 1.47 mm vs. 22.50 ± 2.35 mm, *p *< 0.05). The mean EOA values of the implanted aortic valve were 1.59 ± 0.25 cm^2^ and 1.64 ± 0.19 cm^2^ for ARE-DVR and DVR, respectively (*p *= 0.47). Table 2 summarizes the data on the prosthetic valves.

**Table 2:** Prosthetic valve models and characteristics.

Prosthetic valve size (mm)	Models	EOA (cm^2^)	ARE-DVR *n* (%)	DVR *n* (%)	Total *n* (%)
Aortic					
17	SJM HP	1.1 ± 0.3	2 (8)	4 (9)	6 (8.6)
18	Medtronic AP	1.5 ± 0.3	5 (20)	4 (9)	9 (13.0)
19	On-X	1.5 ± 0.2	8 (32)	11 (25)	19 (27.5)
19	SJM Regent	1.7 ± 0.2	1 (4)	0	1 (1.4)
20	Medtronic AP	1.7 ± 0.2	5 (20)	3 (6.8)	8 (11.5)
20*	Aspire	—	1 (4)	1 (2.2)	2 (2.8)
21	On-X	1.7 ± 0.4	1 (4)	14 (31)	15 (21.7)
21*	Slimline	—	1 (4)	0	1 (1.4)
21	SJM HP	1.4 ± 0.2	0	2 (4.5)	2 (2.8)
21	CE Magna Ease	1.7 ± 0.3	0	1 (2.2)	1 (1.4)
23	On-X	2.0 ± 0.6	0	3 (6.8)	3 (4.3)
25	On-X	2.4 ± 0.8	1 (0)	1 (2.2)	2 (2.8)
Mitral					
23	On-X	2.0 ± 0.6	1	1	2 (2.8)
25	On-X	2.2 ± 0.9	20	36	56 (81.1)
27	On-X	2.2 ± 0.9	2	4	6 (8.6)
27	CE-Perimount	1.8 ± 0.4	0	1	1 (1.4)
29*	Aspire	—	1	2	3 (4.3)
31	On-X	2.2 ± 0.9	1	1	2 (2.8)

EOA, effective orifice area; SJM, St. Jude Medical Hemodynamic Plus; AP, advance performance; CE, Carpentier Edwards.

* EOA not available.

### 3.1. Operative and late outcomes

**Figure 2:**
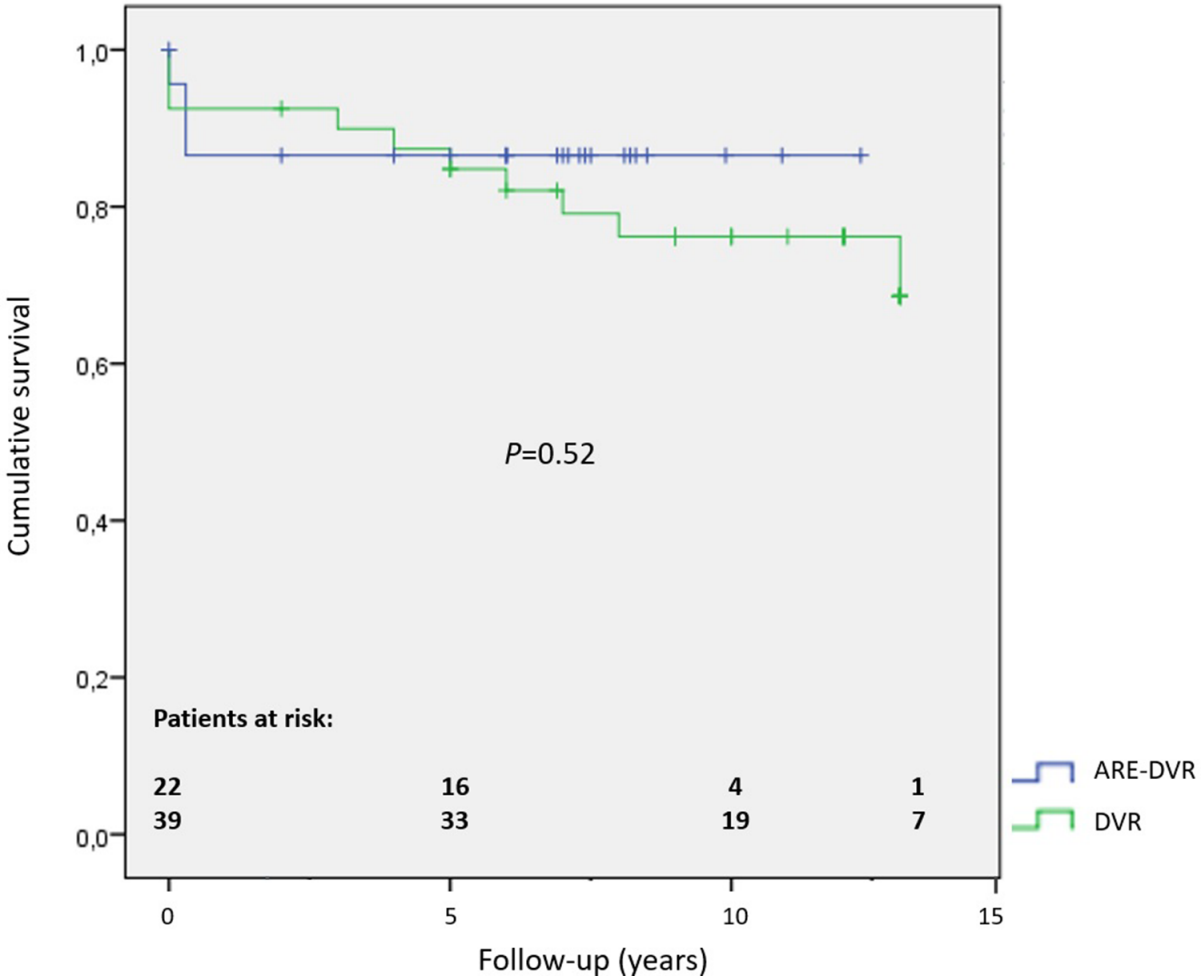
Kaplan–Meier estimates of survival at follow-up.

The aortic cross-clamping duration was greater in the ARE-DVR group (177.6 ± 37.9 min vs. 148.3 ± 66.3 min, *p *= 0.047). The operative mortality rate was 5.6% for the entire cohort, and no statistically significant difference was found between the groups (ARE-DVR: 8% vs. DVR: 4.5%, *p *= 0.46) (Table 3). Table 4 summarizes the clinical data of the deceased patients. The postoperative mean aortic gradient was 19.68 ± 7.20 mmHg (range: 6.14–24 mmHg), with no statistically significant differences between the groups (ARE-DVR: 16.2 ± 9.9 mmHg vs. DVR: 17.3 ± 6.6 mmHg, *p *= 0.62). Table 5 reports the post-operative events according to age. At a mean follow-up of 6.9 ± 3.9 years, the estimated 5-year survival rates for ARE-DVR and DVR were 86.5 ± 7.2% and 89.9 ± 4.8%, respectively (*p *= 0.52) (Figure 2). Only one patient in the ARE-DVR group had undergone reoperation at follow-up for prosthetic valve endocarditis (*n* = 1/22, 4.5%).

**Table 3:** Operative data and early clinical outcomes.

Variables	ARE-DVR (*n* = 25)	DVR (*n* = 44)	Total	*p-*value
Mechanical prostheses, *n* (%)				
Aortic	24 (96.0)	42 (95.4)	66 (95.6)	
Mitral	24 (96.0)	42 (95.4)	66 (95.6)	
Associated procedures, *n* (%)	12 (48.0)	18 (40.9)	30 (43.4)	0.34
Tricuspid surgery	10 (40.0)	17 (38.6)	27 (39.1)	
Myectomy for HOCM	1 (4.0)	0 (0.0)	1 (1.4)	
AV fistula repair	1 (4.0)	1 (2.2)	2 (2.9)	
Nick’s technique, *n* (%)	24 (96.0)	—		
Nunes	1 (4.0)	—	—	
CPB time (min), mean ± SD	221.2 ± 57.9	195.6 ± 75.8		0.148
X-clamp time (min), mean ± SD	177.6 ± 37.9	148.3 ± 66.3		0.047
Postoperative events, *n* (%)				** **
Bleeding	4 (16.0)	4 (9.0)	8 (11.5)	0.36
Arrhythmias	3 (12.0)	6 (13.6)	9 (13.0)	0.84
Tamponade	1 (4.0)	0 (0.0)	1 (1.4)	* *
LCO	2 (8.0)	3 (4.3)	5 (7.2)	0.85
>24 h intubation	1 (4.0)	1 (2.2)	2 (2.9)	* *
ICU stay >96 h	7 (28.0)	17 (38.6)	24 (34.7)	0.37
Operative mortality	2 (8%)	2 (4.5)	4 (5.7)	0.46

CPB, cardiopulmonary bypass; X-clamp, cross-clamping; ICU, intensive care unit; LCO, low cardiac output; HOCM, hypertrophic obstructive cardiomyopathy; AV, aortoventricular; SD, standard deviation.

**Table 4:** Data of the four patients who died during the admission.

** **	A**RE-**DVR		DVR	
	Patient 1	Patient 2	Patient 3	Patient 4
Age	24 years	32 years	45 years	24 years
Gender	F	M	M	F
Preop NYHA class	IV	IV	IV	IV
Cardiac rhythm	Sinus	Sinus	Sinus	Sinus
LVEF	30%	40%	66%	55%
Mitral lesions	SMR	Mixed	SMR	SMR
Aortic lesions	SAR	SAR	SAR	SAR
Tricuspid lesions	STR	None	MTR	MTR
Cardioplegia	Custodiol	Custodiol	Custodiol	Custodiol
ECC time (min)	250	246	208	128
Mech ventilation (h)	22	30		74
ICU length of stay (h)	114	121	184	76
Cause of death	LCO	LCO, tamponade	PE	LCO

NYHA, New York Heart Association; LVEF, left ventricle ejection fraction; ECC, extracorporeal circulation; ICU, intensive care unit; SMR, severe mitral regurgitation; SAR, severe aortic regurgitation; STR, severe tricuspid regurgitation; MTR, moderate tricuspid regurgitation; LCO, low cardiac output; PE, pulmonary embolism.

**Table 5:** Postoperative events according to age.

Variables	ARE-DVR			DVR		
	<20	20–29	>30	<20	20–29	>30
Patients, *n* (%)	11 (44.0)	7 (28.0)	7 (28.0)	14 (31.8)	16 (36.3)	15 (34.0)
Prosthesis EOA, mean ± SD	1.42 ± 0.22	1.58 ± 0.10	1.73 ± 0.28	1.54 ± 0.19	1.63 ± 0.16	1.68 ± 0.16
Operative mortality	0	1	1	0	1	1
Complications^a^	3	4	1	4	5	4

^a^ Bleeding, LCO, arrhythmias, and tamponade.

### 3.2. Discussion

PPM is caused by an inadequacy between the prosthesis orifice area and the body size of the patient. When severe, PPM is associated with increased cardiac events such as poor regression of the left ventricular mass and reduced survival (3). Although PPM occurs in all cardiac valves, it is more common after AVR, with a reported prevalence rate between 20% and 70% (3). Smaller aortic annular size, increased BSA, and younger age at surgery were found to be associated with a major risk of PPM (11, 12).

Although other preventive measures, such as the use of aortic sutureless valves, have progressively gained interest, ARE techniques have historically been advocated to avoid PPM. Castro et al. (13) reported a reduction in the incidence rate of PPM to 2.5% (compared with 17%) in 657 patients who underwent combined ARE and AVR, with no increase in operative mortality. In the largest comparative study by Rocha et al. (14), ARE-AVR and AVR patients had similar postoperative mortality outcomes despite the longer cardiopulmonary bypass (CPB) and X-clamping times in the ARE-AVR group. However, higher in-hospital mortality was found in patients who underwent ARE-AVR when other procedures (coronary bypass or other valve surgeries) were performed. This was corroborated by a recent meta-analysis that reported increased mortality in patients who underwent ARE-AVR in combination with other cardiac procedures (8, 15). In a cohort of 13,174 patients, Sà et al. (8) reported an increase in perioperative mortality in patients who underwent ARE-AVR associated with other procedures compared with those who underwent AVR and associated procedures alone. A successive meta-analysis of 40,447 AVR cases by the same authors confirmed previous findings, with a higher mortality rate in the ARE-AVR and concomitant procedures groups (*p *< 0.001) (15). Although this increased mortality is potentially related to patient factors rather than to ARE, the reluctance to associate ARE techniques with complex surgeries is understandable. Although ARE has been used for more than four decades, experience with such techniques remains poor globally and has been potentially associated with adverse procedural events such as bleeding, patch rupture, and death. Indeed, ARE has been reported in only 5.7%–26.3% of patients undergoing AVR in meta-analyses (8). Moreover, the controversial benefits of ARE in some groups and the growing interest in other techniques, such as transcatheter aortic valve replacement (TAVI) and sutureless valves, have contributed to limiting the need for ARE. In fact, TAVI and sutureless prostheses might be preferable in elderly patients with comorbidities undergoing concomitant procedures rather than a more time-consuming ARE-AVR (16, 17). This re-emphasizes the need to tailor PPM preventive strategies to specific cases, considering the physician's skills, device availability, patient clinical characteristics, and local context.

ARE is commonly indicated in sub-Saharan African (SSA) patients undergoing AVR (36.2% in our series). In fact, a large number of patients, including those with multiple valve diseases, present at surgery with a hypoplastic annulus due to their younger age and small BSA. In this subgroup, biological options, such as stentless or sutureless valves, are limited by early structural deterioration and prohibitive costs. Although technically challenging, the Ross procedure remains a valuable tool because it allows for the growth potential of the neo-aortic valve. However, it is less suitable for cases with multiple valvular lesions, and a high long-term failure rate has been reported in patients with rheumatic diseases (18–20).

To our knowledge, this is the first study to report ARE and double DVR in SSA. We routinely perform aggressive ARE in young patients requiring AVR or DVR by implanting adult-sized prostheses whenever possible, as the potential for growth in these patients remains a determining factor for the late recurrence of PPM. In addition to ARE, high hemodynamic profile prostheses (21) (Medtronic Advance Performance, SJM Hemodynamic Plus, or Regent) were preferred in our aortic patients, representing 42% of the implanted valves. NT has been our preferred ARE technique. When used with the appropriate technique, NT is associated with excellent root stability in the long term, even with an autologous pericardial patch (22). All patients received a larger aortic prosthesis, and no technical difficulties regarding prosthesis implantation were reported despite concomitant adult-size mitral prostheses. We believe that NT is less time-consuming than Manougian's technique. The latter might require a deeper extension in the anterior mitral leaflet and dome of the left atrium, with extensive patch reconstruction time. Despite the higher procedural duration in ARE-DVR patients, no significant differences were found in operative mortality between the two groups. Similar findings were reported by Okuyama et al. (23) and Zhong et al. (24), suggesting that ARE is not an independent factor for mortality in DVR despite the increased operative time. This contrasts with some meta-analyses that reported increased perioperative mortality in patients undergoing ARE-AVR and associated cardiac procedures (15, 15). It is possible that differences in patient demographics and clinics between the current and previous studies led to the observed heterogeneous outcomes. While our study was performed in an SSA environment with younger patients with Rheumatic Heart Diseases (RHD), the patients in the meta-analyses were from Western countries, were older, and presented with degenerative etiologies, including coronary diseases, and high comorbidity rates. As stated earlier, the strategy for PPM prevention, including the indication for ARE, should be tailored to the specific case following an exhaustive assessment of patient characteristics, disease patterns, and team expertise.

The small number of cohorts that have potentially impacted the statistical power of our analysis is a limitation of this study.

In conclusion, the association of the ARE technique with DVR does not significantly affect operative mortality in young SSA with RHD. ARE can be safely used whenever indications arise to reduce the occurrence of PPM and the risk of reoperation, especially in patients with growth potential.

## Data Availability

The raw data supporting the conclusions of this article will be made available by the authors, without undue reservation.
